# DNA aptamer against EV-A71 VP1 protein: selection and application

**DOI:** 10.1186/s12985-021-01631-y

**Published:** 2021-08-12

**Authors:** Xinran Zou, Jing Wu, Jiaqi Gu, Li Shen, Lingxiang Mao

**Affiliations:** 1 Department of Laboratory Medicine, The Affiliated People’s Hospital, Jiangsu University, Zhenjiang City, Jiangsu Province China; 2 Department of Laboratory Medicine, The Affiliated Hospital of Southwest Medical University, Luzhou City, China; 3 Department of Laboratory, Zhenjiang Center for Disease Control and Prevention, Zhenjiang City, Jiangsu Province China

**Keywords:** Aptamer, EV-A71, SELEX, Detection, Therapy

## Abstract

**Background:**

Enterovirus 71 (EV-A71) is a highly infectious pathogen associated with hand, foot and mouth disease, herpangina, and various neurological complications, so it is important for the early detection and treatment of EV-A71. An aptamer is a nucleotide sequence that screened in vitro by the technology named systematic evolution of ligands by exponential enrichment technology (SELEX). Similar to antibodies, aptamers can bind to the targets with high specificity and affinity. Besides, emerging aptamers have many advantages comparing with antibodies, such as ease of synthesis and modification, having a wide variety of target materials, low manufacturing cost and easy flexibility in amending. Therefore, aptamers are promising in virus detection and anti-virus therapy.

**Methods:**

Aptamers were selected by SELEX. Specificity, affinity and second structure were used to characterize the selected aptamers. Chemiluminescence was adopted to build an aptamer-based detection method for EV-A71. Cytopathogenic effects trial, the level of intracellular EV-A71 RNA and protein expression were used to evaluate the antiviral effect of the selected aptamers.

**Results:**

Three DNA aptamers with high specificity and affinity for EV-A71structual protein VP1 were screened out. A rapid chemiluminutesescence aptamer biosensor for EV-A71 detection was designed out. The selected aptamers could inhibit the RNA replication and protein expression of EV-A71 in RD cells and ameliorate the cytopathogenic effects.

**Conclusions:**

The aptamers against EV-A71 have the potentiality to be applied as attractive candidates used for EV-A71 detection and treatment in the future.

## Introduction

Enterovirus 71 (EV-A71) is one of the main pathogens of infective diseases such as hand, foot and mouth disease (HFMD), herpetic pharyngitis, acute flaccid paralysis, encephalitis and so on. Because of highly contagious ability of EV-A71, there have been a number of large-scale epidemics of EV-A71 in many countries and regions [1]. Although most of clinical EV-A71 infections are self-limited diseases, sometimes EV-A71 can cause severe infection and seriously threaten the health of children because of their affinity to the nervous system [2]. At present, the detection of EV-A71 mainly depends on fluorescence quantitative PCR. However, the process of detection is complicated and time-consuming, and the cost is high. The treatment of EV-A71 infection is limited to symptomatic support treatment, lack of special antiviral drugs for EV-A71. There are only a few broad-spectrum antiviral drugs in the treatment of EV-A71 infection, but the curative effect is not ideal [3]. Therefore, it is of great significance to propose a simple and accurate method for EV-A71 detection and develop anti-EV-A71 agents for EV-A71 specific treatment.

EV-A71, a member of the family Picornaviradae, is a non-enveloped, single stranded RNA human enterovirus [4, 5]. Structural proteins of EV-A71 contain VP1, VP2, VP3 and VP4, among which the capsid protein VP1 is the most external part and occupies the predominant protein proportion on the EV71 surface. VP1 plays a significant role in the process of virus infection because it could distinguish and catch cell receptors and assist the virus entry. At the same time, VP1 possesses the main epitopes recognizing EV-A71 neutralizing antibody, which is significant for the immunity of EV-A71. Just as important, these epitopes are very conserved [6], which make VP1 become the hot research subject in diagnose and therapy of EV-A71 [7].

An aptamer is a nucleotide sequence that screened in vitro by the technology named systematic evolution of ligands by exponential enrichment (SELEX) [8]. Similar to antibodies, aptamers can bind to their targets with high specificity and affinity. However, as nucleotide aptamers are easier to synthesize and modify, and have a wider range of target materials, aptamers are promising in virus detection and anti-virus therapy [9]. Currently, there is no DNA and RNA aptamer against EV-A71 proteins or EV-A71 particle has been successfully selected. Considering that VP1 plays a vital role in life cycle of EV-A71, we chose EV-A71 VP1 protein as the target material for screening the aptamer of EV-A71. Meanwhile, we chose Coxsackievirus A16 (CA16) for reverse screening in consideration of the similarity between CA16 VP1 and EV-A71 VP1 [10, 11]. In our research, we screened out three DNA aptamers V7, V11, V21 with high affinity and specificity against EV-A71 VP1. Based on this, using V11 and V21, we built a rapid chemiluminutesescence aptamer biosensor (aptasensor) which was fast and easy to be automated, and then, we confirmed that these aptamers have anti-infecting effect on EV-A71 in vitro.

## Materials and methods

### SELEX procedure

The ssDNAs with random middle sequences of 35 nucleotides (nt) and fixed sequence of 20nt at both ends formed the initial library. Two primers participated the PCRs. The DNA library and primers were provided by Sangon Biotechnology (Shanghai, China). The detailed sequences are showed in Table 1.

**Table 1:** Initial DNA library of SELEX and primers

Name	Sequence	Decoration
library	atccagagtgacgcagca-40n-tggacacggtggcttagt	5′-Biotin
P1-p	atccagagtgacgcagca	5′-Phosphate
P2-b	actaagccaccgtgtcca	5′-Biotin

A, adenine; g, guanine; c, cytosine; t, thymine; n random base

Before the SELEX, the initial nucleotide library was amplified and denatured at 95 ℃ for 5 min. The library was firstly incubated with EV-A71 VP1 protein (50 pmol) at room temperature for 30 min. By incubated with His-tag magnetic beads (MBs), the sequences that had stuck to VP1 would be collected after magnetic concentration. Midazole (500 mM) was applied to separate the MBs and the ssDNA-protein conjugates. Then the supernatant that contained candidate aptamers was amplified by PCR for the next round selection. After undergoing denaturation, purification and delinking, this above-mentioned ssDNA pool turned into new ssDNA library for the next SELEX round. From the 2nd round of SELEX selection, empty MBs and CA16 were separately used for reverse screening to increase the specificity of the library before the library was incubated with EV-A71 VP1 protein. The following steps were same to the first round until the 9th round. The optimal PCR product was cloned in *Escherichia coli* (*E. coli*) BL21 cells. Individual colonies were selected randomly and appraised by PCR. The clone about 232 bp was picked up for sequencing (Sangon, Shanghai, China) [12–14].

### Assessment of the specificity and affinity of aptamers by ELISA

Protein was diluted to the appointed concentration and coated on the 96-well plate at 4 ℃ overnight. Ample washing buffer (1 × PBS buffer, 5 mM MgCl2, 0.02% tween-20) was used to rinsing the plate and 1% BSA (Bovine serum albumin) was used to block the plate for 1 h. After the second washing of the plate, aptamers (5′-biotin) were diluted by binding buffer (1 × PBS buffer, 5 mM MgCl2, 1% BSA, 1 μg/mL tRNA, 0.02% tween-20) to target concentration and incubated with the coated plate for 1 h at room temperature. The wells were washed before streptavidin horseradish peroxidase (SA-HRP, Sangon, Shanghai, China) was diluted with washing buffer (1:200) and added to the plate. The free SA-HRP in each well was removed by washing and 3,3′,5,5′-tetramethylbenzidine (TMB) and H_2_SO_4_ was used to cause the color reaction. The result was interpreted at 450 nm and showed in OD value. The date was analyzed with software Origin 8.0. And the binding curves were plotted according to the equation Y = BmaxX/(*Kd* + X) (X is concentration of aptamer, Y is OD value) and the *Kd*s were calculated from the curves [12].

### Assembly of immunomagnetic beads

Ten microliters MBs dilution (50 mg/mL) was activated with 10 mg/mL ethyl-(dimethylaminutesopropyl)-carbodiimide (EDC, Sigma-Aldrich, Munich, Germany) in pre-cooled 0.4M 2-(N-morpholino) ethanesulfonic acid (MES, Sigma-Aldrich, Munich, Germany) buffer for 30 min at room temperature. Then the supernatant was removed and the aptamer dilution was added to incubate with activated MBs (COOH modified, 1 μm in diameter, Biomag, Wuxi, China) for 6 h at room temperature. The MBs were separated from the supernatant and blocked by blocking buffer (Biomag, Wuxi, China) for 1.5 h at room temperature. Finally, the MBs were washed 3 times with washing buffer and stored in washing buffer at 4 ℃. All incubation of MBs should be kept in light vibration so that the MBs could scatter adequately in the dilution [15, 16].

### Flow cytometry analysis for the confirmation of the connection of MBs and aptamer

The aforementioned immunomagnetic beads were incubated with the complementary nucleotide sequence of V21 (-FAM) at 95 ℃ for 5 min and cooled on ice for 10 min. The MBs were collected by magnetic separation and washed 3 times with washing buffer. Two hundred microliters of binding buffer was then used to resuspend the MBs. The connection of MBs and aptamers was confirmed by detection the fluorescence of MBs with flow cytometry analyzer [15].

### Chemoluminutesescence method for detection EV-A71 based on immunomagnetic beads and aptamers

One hundred and fifty microliters sample solution was incubated with 2 μL of the immunomagnetic beads prepared as above at room temperature for 20 min. After magnetic separation, the MBs were washed 3 times with washing buffer. One hundred microliters of aptamer solution (2.5 nM aptamer in binding buffer) was added to incubate with the MBs-virus compositions at room temperature for 5 min. Subsequently, the complex was washed three times with washing buffer, and 100 μL of SA-HRP in washing buffer (1:1000) was added for 15 min at room temperature. Following by washing as before, the conjugates were resuspended with 100 μL of washing buffer. The chemiluminutesescence light (CL) intensity was measured immediately after 100 μL of substrate solution A and B added into the complex respectively. All incubation of MBs should be kept in vibration [17, 18].

### Cells and virus

Human rhabdomyosarcoma (RD) cells were purchased from the National Collection of Authenticated Cell Cultures (China) and were cultured in Dulbecco's modified Eagle's medium (DMEM, Gibco, Grand Island, USA) with 10% fetal bovine serum (FBS, Gibco, Grand Island, USA). Cells were infected with EV71 at multiplicity of infection (MOI) of 0.01 for 1 h. Then, cells were washed by PBS buffer to remove free virus and further cultured in the DMEM mentioned above. The cells were cultured at 37 °C and 5% CO_2_ [19].

### In vitro antiviral assay

For the antiviral assay, 2 μM aptamer candidate V11, v21, random ssDNA and PBS buffer was added to EV-A71 respectively and incubated at room temperature for 30 min. Before incubation, the library and aptamers should be denaturated at 95 ℃ for 5 min and kept at 4 ℃ [20]. Then, RD cells were incubated with these treated EV-A71 solutions (0.01MOI) respectively at 37 ℃ for 1 h. Next, culture media was renewed to remove the free virus and cells were kept in 37 ℃. The growth state and cytopathic effect (CPE) of the infected cells were observed under microscope after 72 h, the EV-A71 mRNA level of the infected cells was measured by qRT-PCR at 24 h post-infection and the VP1 protein was analysed by western blot at 48 h post-infection.

### Quantitative reverse transcription PCR (qRT-PCR) for detecting EV-A71 mRNA

Total RNA was extracted from virus solution or cultured cells with RNA-Quick Purification Kit (Yishan Biotechnology, China) according to the manufacturer's instructions. The concentration of extracted RNA was detected by NanoDrop spectrophotometer (Thermo Fisher Scientifics, Waltham, USA). For the reverse transcription action, 1000 ng of total cellular RNA was reversely transcribed with a PrimeScript RT reagent kit (Takara, Kyoto, Japan). QRT-PCR analysis was performed with Luminutesaris Color HiGreen qPCR Master Mix (Thermo Fisher Scientifics, Waltham, USA). GAPDH mRNA (Sangon, Shanghai, China) played as an internal control for assessing the expression of cellular RNA [21]. Four hundred nanogram of total RNA of virus solution was used for reverse transcription. The rest of the qRT-PCR steps for pure virus solution were the same as the part of cells’. The primer sequences for EV-A71 were forward, 5ʹ-AGGATTTACATGAGAATGAAGCA-3ʹ, and reverse, 5ʹ-GCATAATTTGGGTTGGCTTT-3ʹ [22].

### Western blot (WB) for detecting the expression of EV-A71 VP1 protein in RD cells

RD cells seeded in 24-well plate were free from the bottom of the wells under the effect of trypsin. Then cells were collected and lysed with RIPA lysis buffer (Kangwei Century, Beijing, China). 12% SDS–polyacrylamide gel was used to separate the extract protein in electrophoresis. Subsequently, the protein was transferred onto polyvinylidene fluoride membranes. The membranes were blocked with 5% nonfat dry milk in 1 × tris-buffered saline containing tween (TBST) buffer for 1.5 h at room temperature on shaking table (150 rpm). Mouse anti-human EV-A71 VP1 monoclonal antibody being provided by the Jiangsu Centre of Disease Control and Prevention was used as the first antibody and mouse anti-β-actin antibody (Sangon, Shanghai, China) was applied as the internal control. Goat anti-mouse HRP-conjugated IgG (Sangon, Shanghai, China) played as the second antibody. Finally, enhanced chemiluminutesescence (ECL) substrates were used to visualize the proteins in according with the manufacturer's directions.

### Statistical analysis

The data processing analysis and graphical plot were finished with the GraphPad Prism version 5.0 software. The data were presented as means ± SD. The analysis of the difference between groups adopted ANOVA. When *P* ≤ 0.05, the difference is considered statistically significant.

## Result

### Selection of candidate aptamers for EV-A71 VP1

From the second round of screening, we added negative screening to increase the specificity of positive screening products, collecting the products of negative screening and positive screening respectively, and amplifying these products by PCR with different cycles, and comparing the products of multiple rounds horizontally and vertically. We found that during the 9 rounds of screening, when the number of PCR amplification cycles was 12, the difference between the electrophoretic signals of the negative and positive screening products of the 8th round screening was the largest. Therefore, the nucleotide product obtained from the 8th round screening was subjected to PCR amplification with 12 cycles for TA cloning and sequencing. Finally, we selected the three sequences with the highest signal and the most repeated copies as candidate aptamers (Table 2).

**Table 2:** Sequences of aptamer candidates specific to EV-A71 VP1

Name	Sequence
V7	actaagccaccgtgtccaUcaaUggUgUgUgcaUUcgUgUgUUgUgUUgUUUgUUgUUUgcUgcgUcacUcUggaU
V11	actaagccaccgtgtccacccUcgccgagUUUUcgUaacUaUaUcUUgUggUUccUaUUgcUgcgUcacUcUggaU
V21	actaagccaccgtgtccaUUcgaUUcgaUcUaaUUUggUUcUUUccUcacUUUUcagUgcUgcgUcacUcUggaU

U, uracil; a, adenine; g, guanine; c, cytosine; t, thymine

### The characterization of candidate aptamers

#### The second structure of candidate aptamers

**Fig. 1:**
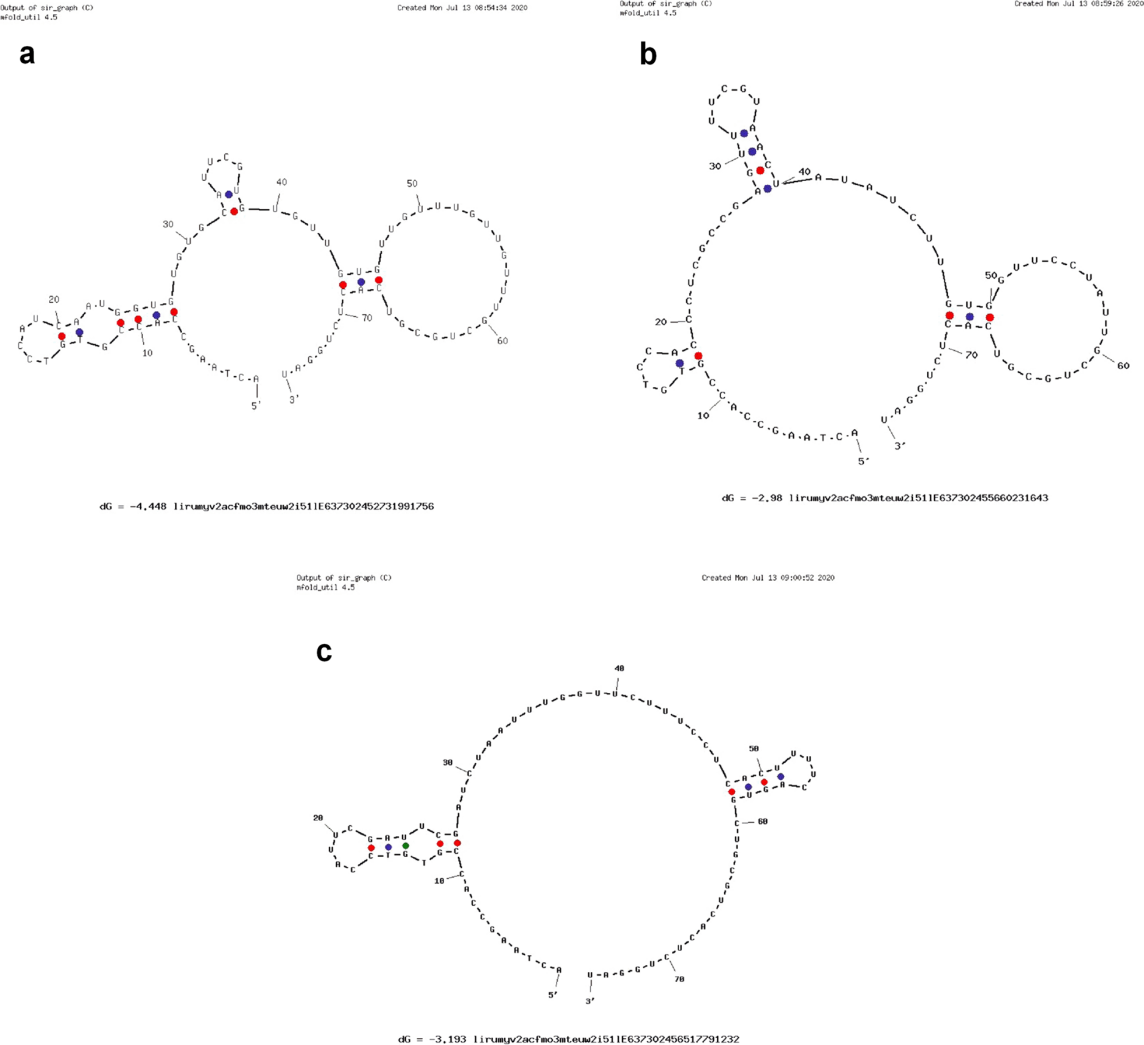
The predicted second structure of aptamer V7, V11, V21. The predicted second structure of aptamer V7 (**a**), aptamer V11 (**b**) and aptamer V21 (**c**)

The second structures of aptamers V7, V11, V21 were predicted by UNAfold tool (Integrated DNA Technologies, America). As showed in Fig. 1, the three aptamers tended to take shape of loop-stem.

#### The specificity of candidate aptamers to EV-A71 VP1 protein

**Fig. 2:**
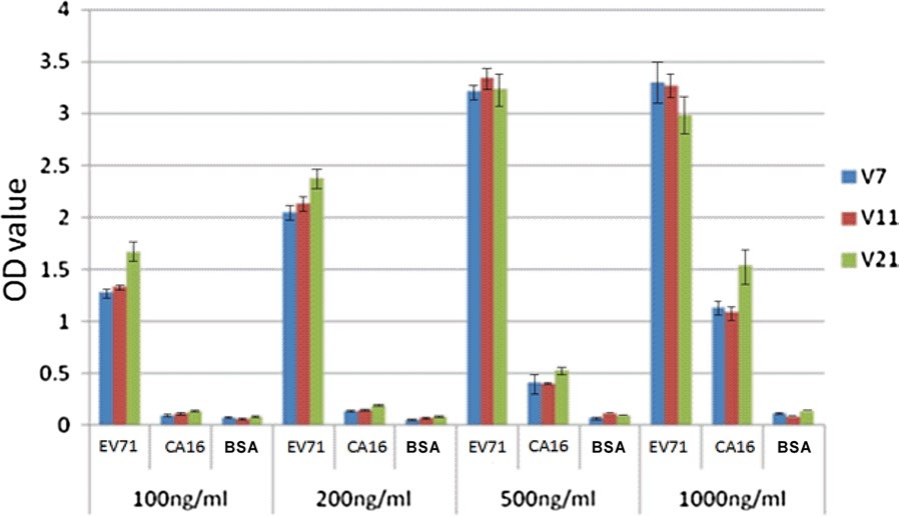
The ELISA for the affinity measurements of three aptamer candidates against EV-A71 VP1 and CA16 VP1 selectivity. BSA played as the negative control (NC). The errors were calculated from three repeated experiments

Enzyme linked immunosorbent assay (ELISA) was used to characterize the specificity of aptamer V7, V11, V21. In which, EV-A71 VP1 protein, CA16 VP1 protein in different concentrations of 100 ng/mL, 200 ng/mL, 500 ng/mL and 1000 ng/mL were planted on 96-well plates. The aptamers (5′-biotin) in 200 nM were used to incubate with these proteins respectively. The result showed, aptamers V7, V11, V21 could fasten with EV-A71 VP1 protein in diverse concentration, while these aptamers did not bind with BSA in each concentration (Fig. 2). And three aptamers bound little with CA16 VP1 protein in the concentrations (≤ 500 ng/mL), only bound to 1000 ng/mL CA16 VP1 protein in a way. Importantly, the binding capacity of the candidate aptamers to CA16 VP1 was evidently weaker than the capacity to EV-A71 VP1 protein. Our result demonstrated the selectivity of aptamers V7, V11, V21 to EV-A71 with no recognition to CA16. In addition, the highest signal-to-noise ratio (SNR) was obtained at the concentration of 200 ng/mL.

#### The affinity of candidate aptamers to EV-A71 VP1 protein

**Fig. 3:**
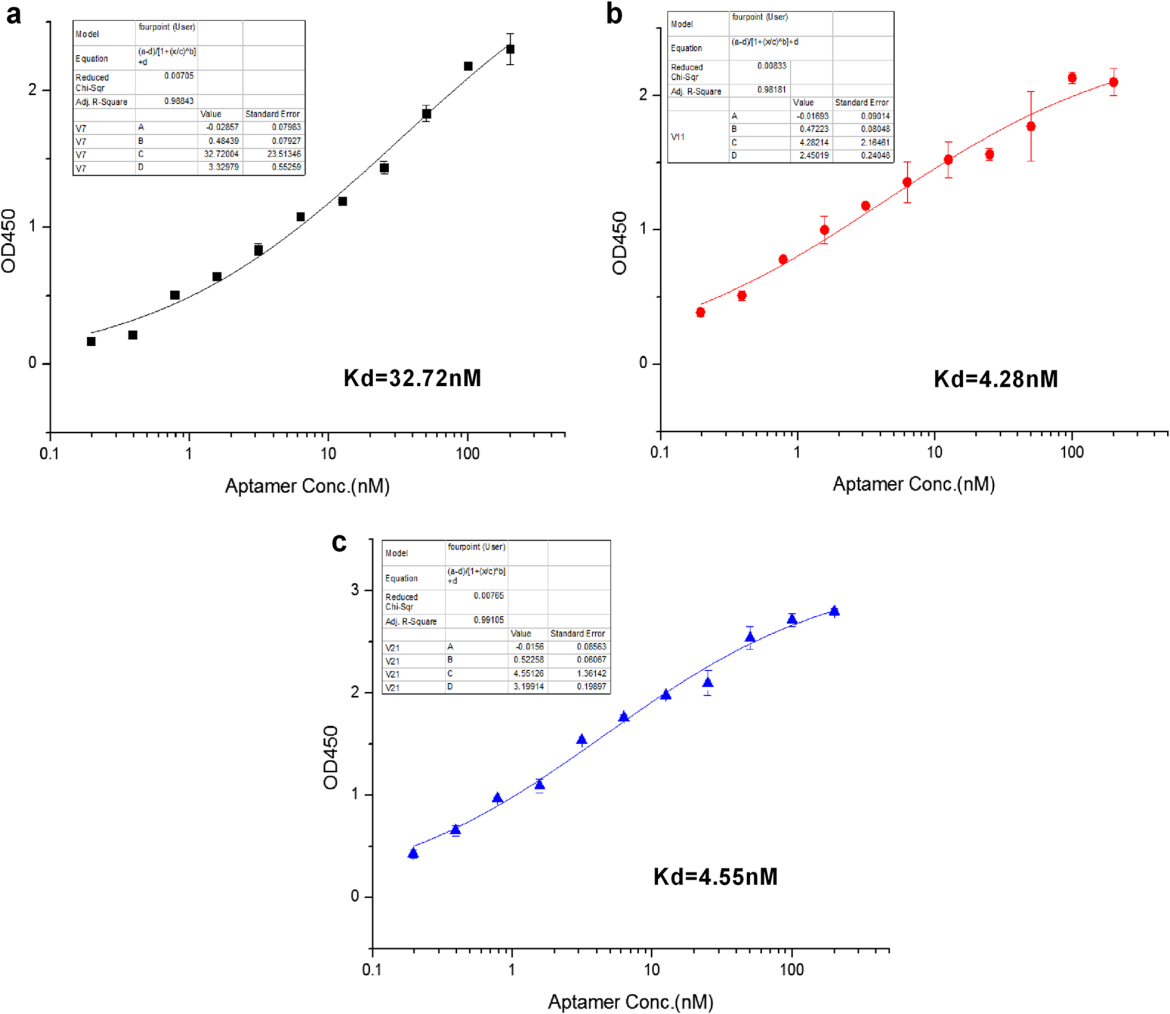
The dissociation constant (*K*_*d*_) values of aptamer candidates for EV-A71 VP1 protein, were measured by ELISA. The *K*_*d*_ value of V7 (**a**). The *K*_*d*_ value of V11 (**b**). The *K*_*d*_ value of V21 (**c**). The values of OD450 and *K*_d_ represent the mean ± SD of three independent experiments

The affinity of the candidate aptamers, expressed in dissociation constant (*Kd*), were also identified by ELISA. Briefly, The EV-A71 VP1 protein in 200 ng/mL was coated on 96-well plates to detect the OD values of aptamers V7, V11 and V21 in different amount. The data was processed with Origin8.0 software and the nonlinear fitting curves were calculated according to the formula Y = Bmax X/(*Kd* + X) (X represents the aptamer concentration and Y represents the OD value). As shown in Fig. 3, the *Kd* values of aptamers V7, V11, V21 were estimated to be about 32.72 nm, 4.28 nm and 4.55 nm, respectively.

### Chemiluminescence (CL) aptasensors for detecting EV-A71

#### The principle of the chemiluminutesescence-based aptasensor for detecting EV-A71

In view of the advantages of aptamer, the potential of aptamer to be used in detection attracted widely attention. Aptasensor means aptamer-based sensor [23]. According to the selecting result, aptamer V7, V11, V11 had similar specificity to EV-A71 VP1 protein, while the affinity of V11 and V21 were much higher than that of V7, so we decided to adopt aptamer V11 and V21 to construct a chemiluminutesescence-based aptasensor for detecting EV-A71.

**Fig. 4:**
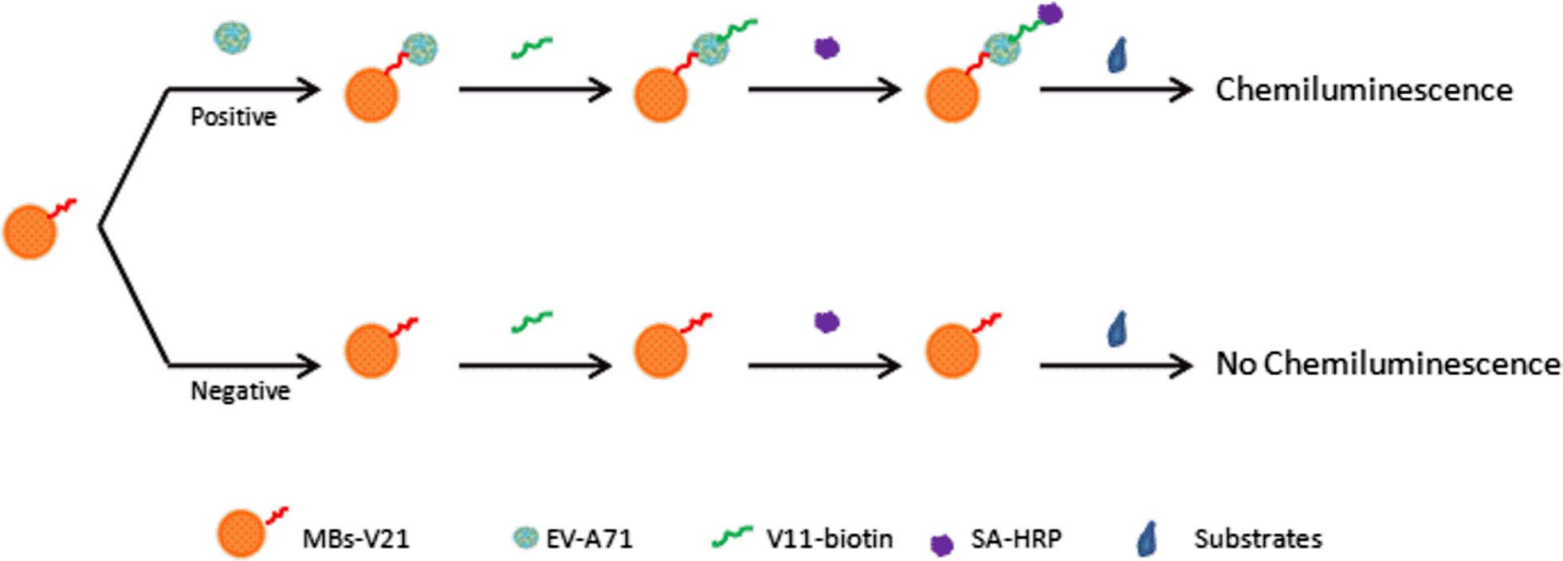
The Schematic illustration of the chemiluminescence aptasensor

As to the principle of the aptasensor (Fig. 4), NH2-V21 was coated on COOH-MBs to build the immunomagnetic beads for capturing the virus. Biotinylated V11 joined to bind with the captured virus, and then the SA-HRP was added to form the complex of the MBs-V21-virus-V11-HRP. Finally, the luminutesescence substrate solution acted on the HRP to excite chemiluminutesescence. If there was EV-A71 in the sample, the chemiluminutesescence value would increase and be positively correlated with the virus content. If there was no EV-A71, the chemiluminutesescence value would be very low.

#### The confirmation of the integration of aptamer and magnetic beads

**Fig. 5:**
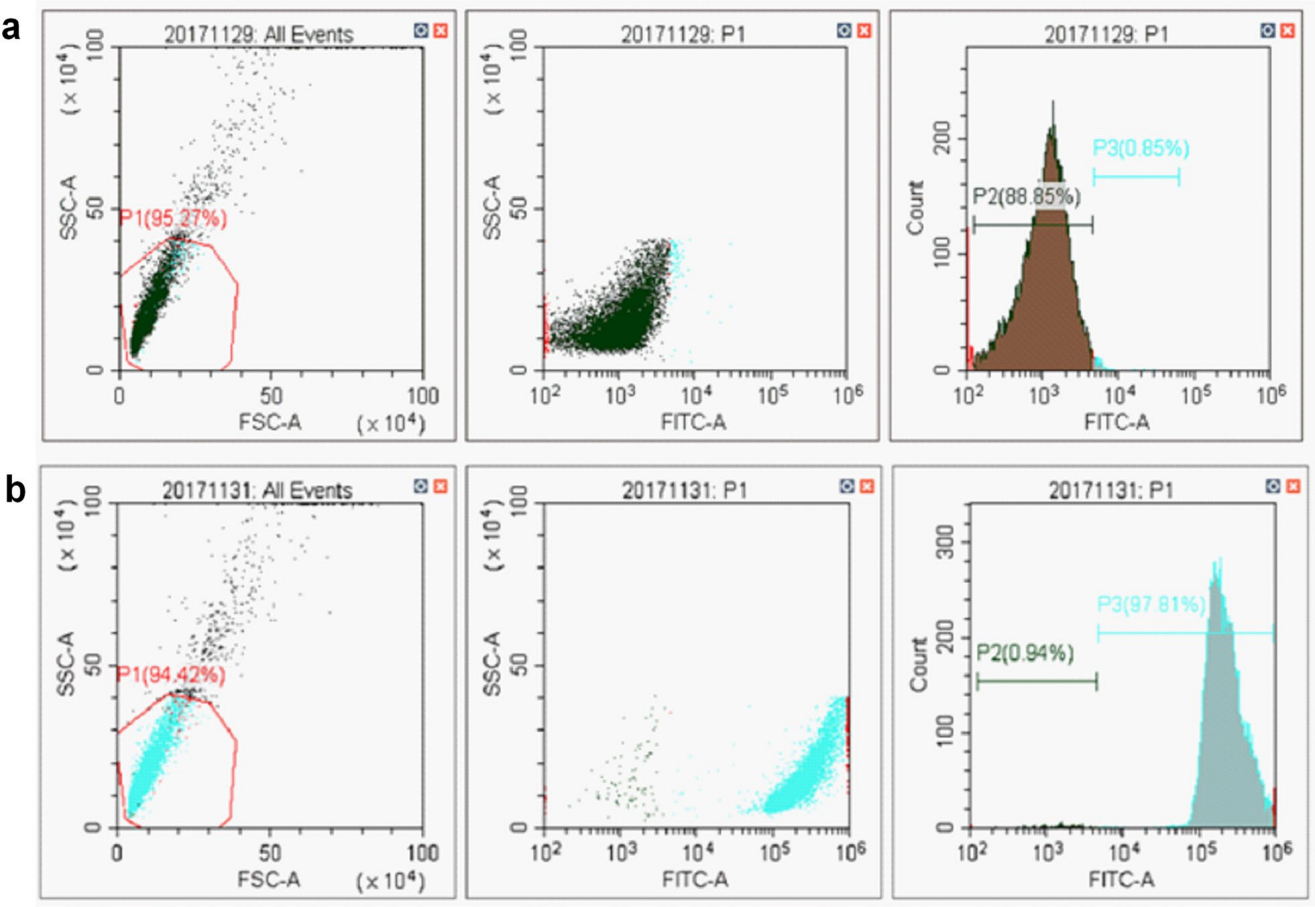
The confirmation of the integration of aptamer and magnetic beads by flow cytometry. Contrast naked MBs (**a**). V21-MBs complex (**b**)

To confirm the integration of aptamer V21 and MBs, we first incubated the V21-coated MBs and bare MBs with complementary sequences of V21 (FAM-tag) respectively. Then, we analyzed the fluorescence intensity of these two groups of MBs by using flow cytometry. The result showed that the fluorescence intensity of the V21-MBs complex group was significantly higher than that of the control group (Fig. 5). It proved that aptamer V21 was successfully coupled to the surface of the MBs.

#### Chemiluminutesescence aptasenser for detecting EV-A71

**Fig. 6:**
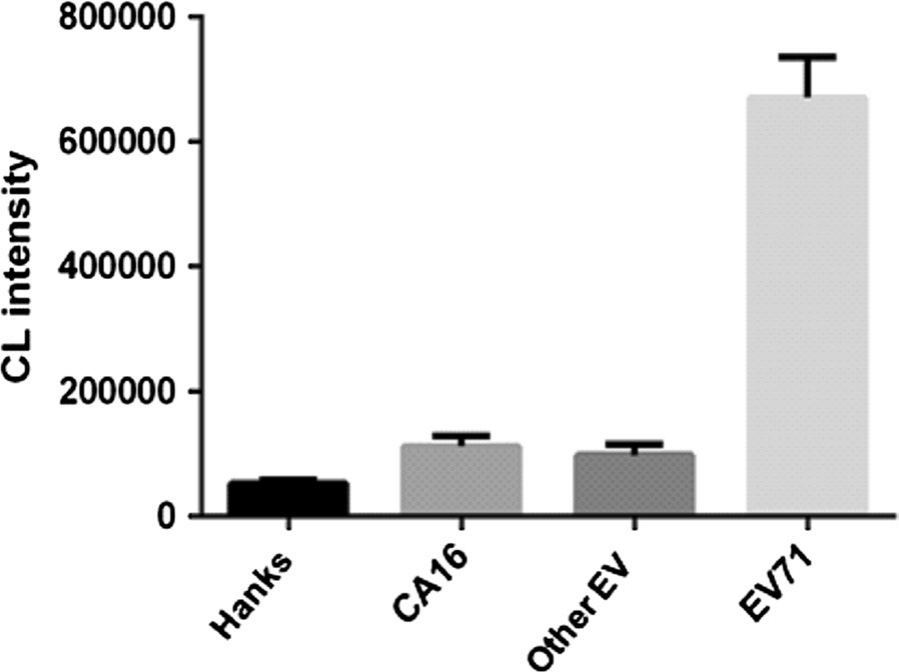
The detection for different samples. Hanks storage solution was adopted as the negative contrast. The values of CL intensity represent the mean ± SD of three independent experiments. Data were obtained from three separate experiments and are presented as the mean ± SD. **Means *P* < 0.001

We made an attempt to measure CA16-positive clinical specimens, other enterovirus-positive specimens and EV-A71-positive clinical specimens respectively. As showed in Fig. 6, the CL intensity of the tested pure Hanks storage solution, CA16 samples, other EV samples were markedly lower than that of EV-A71 samples, which mentions that this aptamer-based detecting method is feasible.

### Feasibility of aptamer in anti-EV-A71 application

**Fig. 7:**
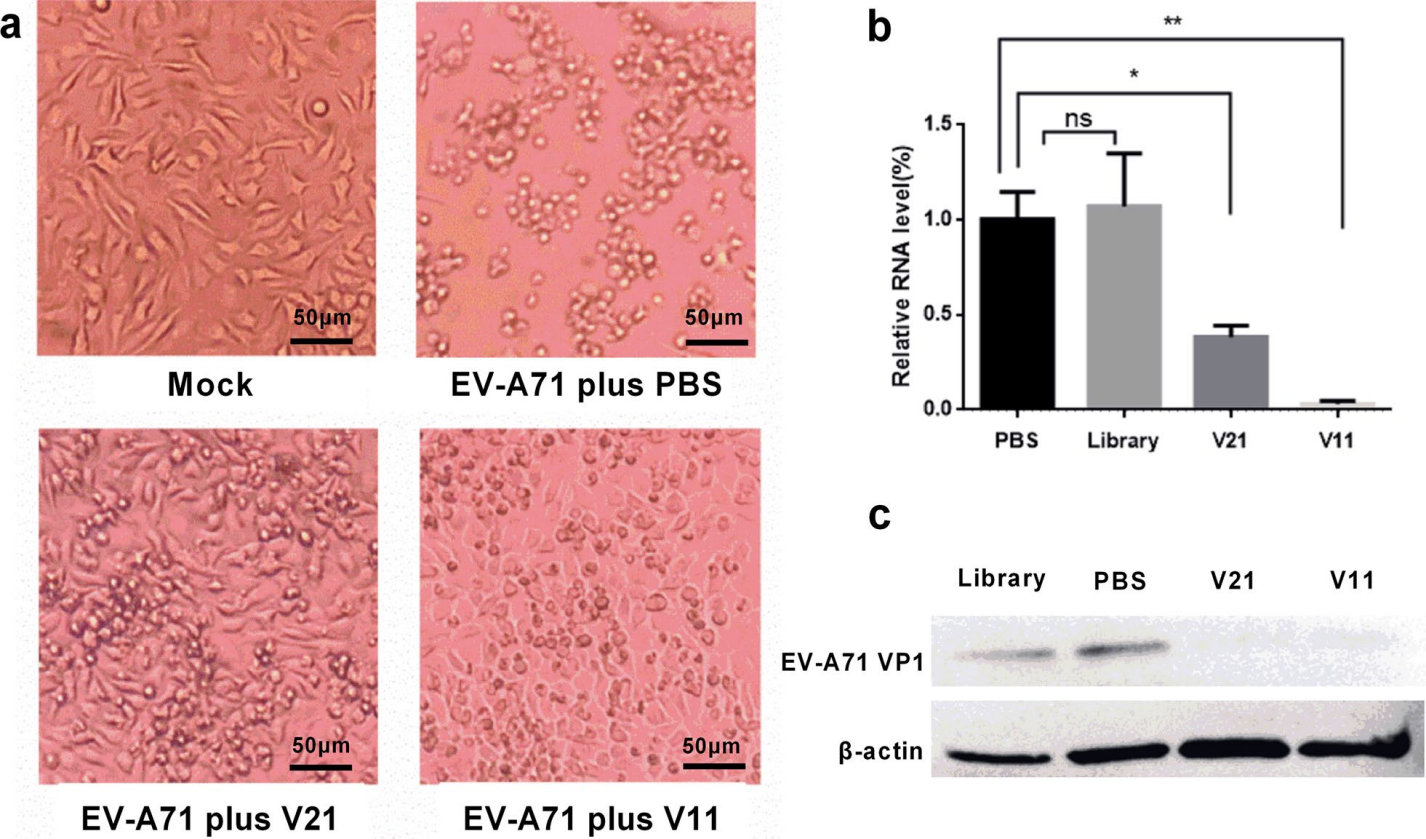
The inhibition of EV-A71 infection by aptamer V11 and V21 in RD cells in vitro. EV-A71 was firstly pretreated by PBS buffer, random ssDNA library, aptamer V11 and V21 respectively at room temperature for 30 min. Then RD cells were infected with treated EV-A71 through incubation at 37 ℃ for 60 min. After renewal of the culture solution to remove free virion and aptamer, the RD cells were cultured at 37 ℃. **a** The CPE of RD cells were observed under optical microscope at 72 h post-infection. The normal untreated RD cells were adopted as the mock group. **b** RT-qPCR analysis was performed to detect EV-A71 mRNA in RD cells infected by original EV-A71 (PBS), random ssDNA library-medicated EV-A71 or aptamer-medicated EV-A71 at 24 h post-infection. **c** The expression of EV-A71 VP1 protein in RD cells infected by EV-A71 with different treatment at 48 h post-infection. The β-actin protein was employed as the internal reference protein. Data were obtained from three separate experiments and are presented as the mean ± SD. *Means *P* < 0.05; **means *P* < 0.001

To explore whether the selected aptamers V11 and V21 had antiviral effects on EV-A71, we performed anti-virus assays in vitro using pre-treated virus of aptamer. RD cells were incubated with diverse pre-treated EV-A71 solutions respectively. In this assay, as showed in Fig. 7a, although compared with the mock group (negative control), the RD cells infected with aptamer-treated EV-A71virus underwent CPE, the degrees of CPE in test groups were obviously lighter than that of the cells infected with PBS-treated EV-A71. The EV-A71 mRNA expression level of the cells treated with the random ssDNA library in RD cells was comparable to that of untreated virus (PBS group), while the mRNA expression levels of the EV-A71 treated with V11 and V21 were significantly lower than that of the PBS group and the library group (Fig. 7b). The expression levels of EV-A71 VP1 protein in the RD cells infected with aptamer-treated EV-A71 virion were observably lower than the cells infected with PBS-treated virus and random ssDNA library-pretreated virus (Fig. 7c). In summary, these results suggested that aptamers V11 and V21 have an inhibitory effect on EV-A71 infection in RD cells.

## Discussion

EV-A71 infection is a serious health problem in the world. But at present, there is no quick and easy detection method and efficient medicine for EV-A71 infection. The emergence of aptamers provides a new idea for the detection and treatment of EV-A71 infection.

Although RNA aptamers usually have higher affinity than their corresponding DNA aptamers, DNA aptamers are much more stable (especially in internal environment) and low-cost than RNA aptamers [24, 25]. So in this research, we chose DNA aptamer instead of RNA aptamer. Now, we have used the EV-A71 VP1 protein to select three DNA aptamers with high affinity and specificity for EV-A71 VP1 through SELEX technology. In order to increase the affinity of aptamers for EV-A71, we used special bases in the process of aptamer selection, although the synthesis cost of deoxynucleotide sequences containing special bases is higher than that of ordinary nucleic acid sequences. The synthesis cost maybe decrease with the development of technology about aptamer. As Fig. 1 showed, the three candidate aptamers spontaneously tended to form hairpin structures or stem-loop structures. These hairpin structures or stem-loop structures tend to hide the sites where the aptamer binds to the target substance. Therefore the aptamers needs to be pre-denaturated in 95 ℃ for 5 min before use [20].

At present, the detection method of EV-A71 infection is mainly nucleic acid detection by PCR. That is time-consuming and needs complex preprocessing. Here, we used two aptamers, V11 and V21, to initially construct a MBs-based EV-A71 chemiluminutesescence detection method. The entire detection process of the aptasensor only takes not more than 40 min, the operation is simple and the detection can be automated, which is very attractive for clinical detection. For the moment, although there is a distinct discrepancy between the CL values obtained between the negative contrast and the EV-A71 virus solution, the sensitivity and other parameters of the method need to be further optimized.

Besides, our research confirmed that the obtained candidate aptamers can inhibit the infection ability of EV-A71, which provided new possibility for the treatment of EV-A71 infection. Instead of working as therapeutics themselves, aptamers are also promising as delivery tools in targeted transportation. Aptamers can be combined with nanoparticles, siRNA/miRNA, and chemical drugs to formulate new therapeutic complexes to achieve the desired therapeutic effect [26, 27]. Based this, it may be served as attractive candidates for EV-A71 antiviral combination therapies in the future.

## Conclusion

In this study, three aptamers with high specificity and affinity for EV-A71VP1 protein were screened by SELEX technology. Using two of the aptamers, an EV-A71 chemiluminutesescence rapid detection method based on MBs was constructed. By observing the CPE, the viral RNA and VP1 protein of EV-A71-infected RD cells after aptamer treatment or not, we found that aptamers V11 and V21 had apparent effect of inhibiting EV-A71 infection in vitro. It proved that the aptamers screened in this study had potential value in the detection and treatment of EV-A71.

## Data Availability

All data supporting the conclusions of this article are included in this published article.

## Abbreviations

EV-A71: Enterovirus 71
SELEX: Systematic evolution of ligands by exponential enrichment technology
HFMD: Hand, foot and mouth disease
CA16: Coxsackievirus A16
MES: 2-(N-morpholino) ethanesulfonic acid
EDC: Ethyl-(dimethylaminutesopropyl)-carbodiimide
SA-HRP: Streptavidin horseradish peroxidase
DMEM: Dulbecco's modified Eagle's medium
FBS: Fetal bovine serum
BSA: Bovine serum albumin
RD cell: Human rhabdomyosarcoma cell
MBs: Magnetic beads
TMB: 3,3′,5,5′-Tetramethylbenzidine
CL: Chemiluminutesescence light
TBST: Tris-buffered saline containing tween
ECL: Chemiluminutesescence
ELISA: Enzyme linked immunosorbent assay
SNR: Signal-to-noise ratio
*Kd*: Dissociation constant
CPE: Cytopathic effect
